# Diagnostic accuracy of the screenings Sniffin’ Sticks Test (SST-12) in COVID-19 induced olfactory disorders

**DOI:** 10.1371/journal.pone.0295911

**Published:** 2024-01-10

**Authors:** Emma J. A. Schepens, Inge Stegeman, Digna M. A. Kamalski

**Affiliations:** 1 Department of Otorhinolaryngology- Head and Neck Surgery, University Medical Center Utrecht, Utrecht, The Netherlands; 2 Brain Center, University Medical Center Utrecht, Utrecht, The Netherlands; University of Catania, ITALY

## Abstract

Objective olfactory function can be assessed using validated olfactory tests like the Sniffin’ Sticks Test (SST). However, their extensive nature makes them less suitable for clinical practice. To address this, shorter olfactory tests like the screenings Sniffin’ Sticks Test (SST-12) can be used for screening purposes and reduce testing time. The SST-12 serves as a diagnostic tool for screening olfaction in cases unrelated to COVID-19. However, these screening tests are uncertain regarding their accuracy in detecting olfactory dysfunction in patients with COVID-19 as the plausible cause. We aim to determine the diagnostic accuracy of the SST-12 in adults with post-COVID-19 olfactory dysfunction. We performed a diagnostic accuracy study with data from 113 consecutive COVID-19 diagnosed patients who experienced objectified smell loss ever since. At approximately 6 months after their diagnosis, all participants underwent the SST (reference standard), part of the SST was the SST-12 (index test). Diagnostic accuracy of the SST-12 is measured as negative predictive value (NPV), positive predictive value (PPV), sensitivity, and specificity. The SST-12 detected smell loss in 85 patients among 91 patients with smell loss and ruled out smell loss in 15 patients among the 22 patients without smell loss based on the reference standard. Making sensitivity 93.4% (CI 0.87–0.97), and specificity 68.2% (CI 0.48–0.85). Out of the 92 patients with a positive test result on SST-12, 85 patients had indeed smell loss (PPV 92.4% CI 0.86–0.97), and out of the 21 patients with a negative test result, 15 patients had no smell loss regarding the reference standard (NPV 71.4% CI 0.50–0.88). The findings suggest that the SST-12 holds promise as a useful tool for identifying individuals with smell loss, also in individuals with COVID-19 as cause, but it is important to have a good understanding of the interpretation of the results of the SST-12 when considering its implementation in clinical practice.

## Introduction

Olfactory dysfunction has emerged as a common symptom in COVID-19. Persistent symptoms can result in a decline of quality of life, affecting nutritional, physical well-being and cognitive functioning [1–4]. Thus, assessing olfactory function accurately and efficiently is essential. Not only for clinical diagnosis and understanding the underlying mechanisms of olfactory dysfunction in COVID-19 patients, but it also enables healthcare professionals to provide patients with objective measurements of symptom severity, and guidance throughout their recovery.

Merely 10% of ENT-surgeons utilizes psychophysical tests for the evaluation of olfaction [5]. Most ENT-surgeons utilize subjective questionnaires, but this approach often yields inconsistent results and potentially leads to underestimation of the extent of the problem [5]. The most utilized psychophysical test in Europe is the validated Sniffin’ Sticks Test® (SST) [5, 6]. The SST provides a comprehensive evaluation of olfactory function, including the ability to identify specific odors, discriminate between different odors, and detect odor thresholds. These type of extended tests are the gold standard for diagnosing olfactory disorders [5, 7]. However, the time-consuming nature of the SST (around 30 to 60 minutes) [8, 9] and the need for sustained concentration from patients make it less suitable for routine clinical practice [10, 11].

In response to the need for more time-efficient olfactory tests, a screening version of the SST, known as the SST-12, has been developed [12]. The SST-12 serves as a diagnostic tool for screening olfaction in causes unrelated to COVID-19. As only 12 scents have to be identified, the test can be done in 5 minutes [13]. The SST-12 focuses solely on the identification subdomain, providing a quick assessment of an individual’s ability to identify twelve odors [14–16]. As the pathophysiology of smell loss varies between COVID-19 and other causes, particularly impacting the threshold domain [17, 18], we explored the SST-12’s ability to also detect smell loss in patients with COVID-19 as the plausible cause [13].

## Methods

### Patients and procedures

In order to assess the diagnostic accuracy of the SST-12, we included 113 consecutive patients. The cohort of participants included in this study originated from the COCOS trial [19]. This was a randomized controlled trial determining the possible benefit of an oral prednisolone treatment (10 days 40mg) on the olfactory function in patients with COVID-19 induced smell disorders. The institutional Review Board of the University Medical Center Utrecht approved the research protocol (21-635/G-D, October 2021). We obtained from all patients written informed consent in order to participate. Patients were recruited via the Dutch media and via the National patients association, between November 2021 and January 2022.

**Fig 1:**
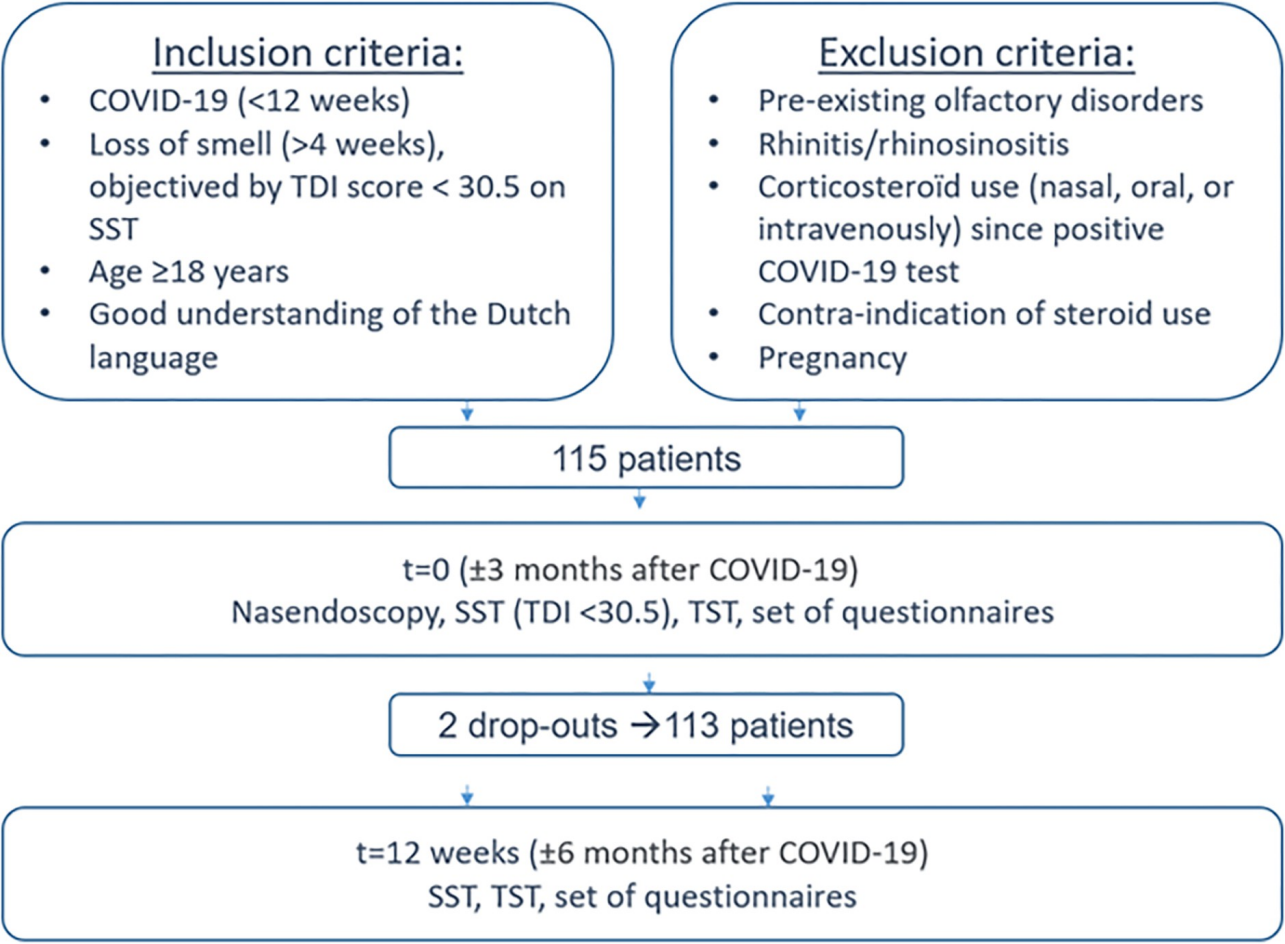
Procedures and participant flow-chart.

[20] Patients approached us by email and consecutive eligible patients were planned for inclusion by telephone. Inclusion criteria were adult patients with good understanding of the Dutch language, with a PCR confirmed COVID-19 diagnosis within 12 weeks before inclusion, and at least 4 weeks of smell loss since their diagnosis. Patients visit the outpatient clinic for Ear, Nose and Throat at baseline for the assessment of the SST and thereby objectify their smell loss. They were excluded when we objectified no smell loss at baseline with SST (TDI score >30.5), or when we found other causes for smell loss objectified by a nasendoscopy, such as nasal polyps or (rhino)sinusitis (Fig 1), or pre-existing smell loss before the COVID-19 diagnosis. These aforementioned criteria make loss of smell due to COVID-19 the most plausible cause. All patients signed informed consent in order to participate. They all performed the Sniffin’ Sticks test again at approximately 6 months after their diagnosis (Fig 1) [20]. Results showed no difference in olfactory function between patients who received prednisolone and those who received placebo [21]. More information about the inclusion- and exclusion criteria and the study procedures of the RCT are described elsewhere [20, 21]. In our analysis, we utilized the results of the SST during the visit approximately 6 months after diagnosis. The reason behind selecting this specific time point is that predictive values are influenced by the prevalence of the disease [22]. By choosing the second visit, we aimed to achieve a well-balanced representation of smell loss prevalence [23, 24].

### Index tests and reference standard

The reference standard is the extended version of the Sniffin’ test® (SST) [25]. The Index test is the SST-12. These Sniffin’ Sticks are manufactured by Burghart, a company with medical certification (ISO 13485). This certification ensures that both the odorants and their solvent used in the test are safe for health, providing confidence in the test’s reliability. We used for both tests the translated version. The SST consists of three subdomains, one of which is the identification test. In the identification subdomain of the SST, patients are presented with 16 different odors and are required to identify the corresponding scent from a set of four options. Out of these 16 odors, 12 are included in the SST-12. So, the moment of assessing the reference standard and index test was at exactly the same moment, ensuring that there was no access to outcome information from one test when assessing the other.

### Statistical analysis

In the sample size calculation, we assumed that more than 50% of the participants would experience a loss of smell. By setting a diagnostic test power of 0.95, a delta of 0.1, a statistical power of 0.8, and a significance level of 0.05, a total of 68 patients was required. All analyses were done in IBM SPSS Statistics 27.0. The reference standard (SST) was used as recommended in the development paper [26], where normosmia is defined as a TDI score >30.5 [27]. Thresholds for the SST-12 were defined as a normosmia (SST-12 ≥ 11). a hyposmia (10 > SST-12 > 6), or an anosmia (SST-12 ≤ 6) [14] In this analysis, we used only the threshold values for hyposmia and normosmia to determine whether a patient possesses a normal sense of smell. We constructed a 2 by 2 table to determine the accuracy and calculated the negative predictive value, positive predictive value, sensitivity and specificity with their confidence intervals. We reported the results according to the STARD guidelines [28].

### Outcome measurements

#### Negative predictive value (NPV)

The NPV is the probability of not having the condition when the test result is negative. This can be calculated by dividing the number of true negative results (TN) by the total number of negative test results. The NPV is important when the aim is to avoid missing cases of smell loss, although there’s a change of false-positive (FP) cases [22]. In our study this is an important outcome, in order to provide guidance to affected patients and because no harmful follow-up diagnostics or treatments are available for false-positive cases. Moreover, the test targets only individuals who self-report smell-loss suspicion, minimizing unnecessary concern which can be the case in random screening.

#### Positive predictive value (PPV)

The PPV is the probability of having the condition when the test result is positive. This can be calculated by dividing the number of true positive results (TP) by the total number of positive test results. The PPV is particularly relevant when the follow-up diagnostic or treatment procedures may have potential harm, costs or other forms of impact [22].

#### Sensitivity

Sensitivity is the measure of a test’s accuracy in medical diagnostics. It represents the percentage of true positive test results (TP) among all diseased individuals. The higher the sensitivity, the greater the likelihood that someone who truly has the disease will receive a positive test result, which is useful when ruling out a disease is desirable. With high sensitivity, there will be fewer false-negative (FN) test results. The sensitivity is calculated using the following formula (TP/(TP+FN) [22].

#### Specificity

Specificity is the measure of true negative test results (TN) among non-diseased individuals. The higher the specificity, the greater the likelihood that someone who does not have the disease will receive a negative test result, which is useful when confirming a disease is desirable. With high specificity, there will be fewer false-positive (FP) test results. The specificity is calculated using the following formula (TN/(TN+FP)) [22].

## Results

Table 1 describes the characteristics and outcome measurements of the SST. Median time in days between conformed COVID-19 and the utilized tests for this analysis is 140 (IQR 128–154). Median TDI score on SST was 27.5 (IQR 23.63–30.0).

**Table 1:** Characteristics and outcome measurements at the moment of performing the SST. Data are presented as median (IQR) or n (%).

	N = 113
**Age, years**	50 (40.5–57)
**Sex**	
**Female**	72 (63.7)
**Male**	41 (36.3)
**Time between confirmed COVID test and test performing, days**	140 (128–154)
**Sniffin’ Stick Test (SST)**	
**TDI score**	27.5 (23.63–30.0)
**Threshold**	4.5 (3.3–5.6)
**Discrimination**	11.0 (10.0–13.0)
**Identification**	11.0 (10.0–13.0)

Table 2 presents the outcomes of the 2 by 2 table analysis.

**Table 2:** Cross-tabulation.

Index test	Reference standard Smell loss	No smell loss	
SST-12 positive	85	7	92
SST-12 negative	6	15	21
Total	91	22	113

Sensitivity was 0.934 (CI 0.87–0.97). Among the 91 individuals with smell loss regarding the reference standard, 85 (93.4%) participants were detected with a positive test result on the SST-12. Specificity was 0.682 (CI 0.48–0.85). Among the 22 individuals without smell loss, 15 (68.2%) participants were ruled out to have smell loss. The PPV of the SST-12 was calculated as 0.924 (CI 0.86–0.97). Among the 92 individuals with a positive test result, 85 (92.4%) participants did have smell loss based on the reference standard. The NPV of the SST-12 was calculated as 0.714 (CI 0.50–0.88). Among the 21 individuals with a negative test result, 15 (71.4%) participants did not have smell loss based on the reference standard.

## Discussion

The aim of this study was to identify the diagnostic accuracy of the SST-12 for COVID-19 induced loss of smell. We found a high PPV 92.4% (CI 0.86–0.97) and sensitivity 93.4% (CI 0.87–0.97), and an acceptable NPV 71.4% (CI 0.50–0.88) and specificity 68.2% (CI 0.48–0.85).

These findings were achieved by a comprehensive cohort design which included a large sample size of consecutive patients enrolled within a concise period. All patients had a confirmed COVID-19 diagnosis by PCR in the same timeframe since performing the SST. Besides we used a predetermined threshold, which contributes in the validity and generatability of the results. To the best of our knowledge, Vandersteen et al. performed the only study that included patients with post COVID-19 olfactory dysfunction to investigate the diagnostic accuracy of the SST-12, but their small sample size and wide confidence intervals raise uncertainty about the applicability of their results [13]. Hummel et al investigated the diagnostic accuracy of the SST-12 and of the Q-sticks (a three-odor test), but in non-COVID-19 related cases [14, 29]. Our results, however are comparable to theirs.

It is important to note that as we followed the typical sequence for all subdomains, from threshold to discrimination and identification, some patients may have experienced decreased concentration during the final identification test. In the SST-12, patients only perform the identification part. While this may yield different results compared to the extended SST’s identification section, as all patients underwent this procedure, we do not expect it to significantly impact our findings.

The high sensitivity in our study indicates that among patients with smell loss, a high number of patients will indeed receive a positive test result, resulting in few false negatives. If the SST-12 had been used in this study, six patients (6.6%) would have received a false-negative test result. The high sensitivity value suggests that the SST-12 is effective in correctly identifying individuals with smell loss and minimizing false-negative test outcomes. However, in the study of Sorokowska et al. there was a high number of false-negatives, but this contradiction in comparison with our study can be found in the fact that they used the Q-sticks, and because they did not use the SST as reference standard [6] (Sorokowska et al., 2019).

We found a moderate specificity, indicating that the SST-12 is amendable for obtaining false positives. In this study, if the SST-12 had been used instead of the SST, seven patients (32%) would have received a false-positive test result. Though, the interpretation and usefulness of any diagnostic test is dependent on the setting in which it is used. Most smell tests will be used in clinical settings, in patients in which more objective knowledge about their ability to smell can be crucial for provide guidance throughout their recovery trajectory. The consequences of the high sensitivity in combination with the relatively low specificity is a possibility of detecting a relative high number of false positives. There are however no harmful or expensive follow-up diagnostic tools or unnecessary treatment options for smell loss when patients test false-positive.

The moderate NPV we found, suggests that there is a possibility of missing the diagnosis. This could be attributed to the fact that the SST-12 only assesses the identification ability, while in COVID-19 patients the threshold domain seems most affected [17, 18]. The reason for this is the fact that of the olfactory threshold assessment primarily relies on the peripheral olfactory system, specifically the olfactory epithelium, which is the part most accessible to the SARS-CoV-2, while the identification and discrimination components are more closely associated with higher-level cognitive processes [30, 31]. These patients may perform well on the SST-12, but still have COVID-19-induced loss of smell, which can only be assessed by the SST. Since COVID-19 induced smell disorders can have a significant impact on individuals’ quality of life, it is advisable to combine the SST-12 with other clinical information to make an accurate diagnosis. In cases where the disease is suspected, even if the SST-12 is negative, additional testing may be necessary. Alternatively, healthcare workers can still provide guidance and support to help patients manage their complaints. The high PPV we found in combination with the high sensitivity makes the SST-12 especially helpful for identifying smell loss. Considering the diagnostic accuracy of the SST-12, it has the potential to aid in early detection and in monitoring disease progression. In general practice the SST-12 will mostly be used for counseling, and to follow the trajectory of the smell function since patients cannot objectify the vague improvement of their smell themselves. Understanding the limitations and potential false results of the test is relevant for managing patient expectations and ensuring appropriate counseling.

## Conclusion

Our findings suggest that the SST-12 holds promise as a screenings tool in identifying smell loss, also in patients with COVID-19 as the most plausible cause.

## References

[1] Vandersteen C, Payne M, Dumas L-E, Metelkina-Fernandez V, Plonka A, Chirio D, et al. Persistent olfactory complaints after COVID-19: a new interpretation of the psychophysical olfactory scores. Rhinol Online. 2021;4: 66–72. doi: 10.4193/RHINOL/21.010

[2] Croy I, Nordin S, Hummel T. Olfactory Disorders and Quality of Life—An Updated Review. Chem Senses. 2014;39: 185–194. doi: 10.1093/chemse/bjt072 24429163

[3] Aschenbrenner K, Hummel C, Teszmer K, Krone F, Ishimaru T, Seo H-S, et al. The Influence of Olfactory Loss on Dietary Behaviors. Laryngoscope. 2008;118: 135–144. doi: 10.1097/MLG.0b013e318155a4b9 17975508

[4] Mai Y, Menzel S, Cuevas M, Haehner A, Hummel T. Well-being in patients with olfactory dysfunction. Physiol Behav. 2022;254: 113899. doi: 10.1016/j.physbeh.2022.113899 35809697

[5] Hummel T, Whitcroft KL, Andrews P, Altundag A, Cinghi C, Costanzo RM, et al. Position paper on olfactory dysfunction. Rhinology journal. 2017;54: 1–30. doi: 10.4193/Rhino16.248 29528615

[6] Sorokowska A, Oleszkiewicz A, Minovi A, Konnerth CG, Hummel T. Fast Screening of Olfactory Function Using the Q-Sticks Test. ORL. 2019;81: 245–251. doi: 10.1159/000500559 31256162

[7] Whitcroft KL, Cuevas M, Haehner A, Hummel T. Patterns of olfactory impairment reflect underlying disease etiology. Laryngoscope. 2017;127: 291–295. doi: 10.1002/lary.26229 27556251

[8] Doty RL. Office Procedures for Quantitative Assessment of Olfactory Function. Am J Rhinol. 2007;21: 460–473. doi: 10.2500/ajr.2007.21.3043 17882917

[9] Su B, Bleier B, Wei Y, Wu D. Clinical Implications of Psychophysical Olfactory Testing: Assessment, Diagnosis, and Treatment Outcome. Front Neurosci. 2021;15. doi: 10.3389/fnins.2021.646956 33815048 PMC8012732

[10] Su B, Bleier B, Wei Y, Wu D. Clinical Implications of Psychophysical Olfactory Testing: Assessment, Diagnosis, and Treatment Outcome. Front Neurosci. 2021;15. doi: 10.3389/fnins.2021.646956 33815048 PMC8012732

[11] Doty RL. Office Procedures for Quantitative Assessment of Olfactory Function. Am J Rhinol. 2007;21: 460–473. doi: 10.2500/ajr.2007.21.3043 17882917

[12] Hummel T, Rosenheim K, Konnerth C-G, Kobal G. Screening of Olfactory Function with a Four-Minute Odor Identification Test: Reliability, Normative Data, and Investigations in Patients with Olfactory Loss. Annals of Otology, Rhinology & Laryngology. 2001;110: 976–981. doi: 10.1177/000348940111001015 11642433

[13] Vandersteen C, Payne M, Dumas L-É, Plonka A, D’Andréa G, Chirio D, et al. What about using sniffin’ sticks 12 items test to screen post-COVID-19 olfactory disorders? European Archives of Oto-Rhino-Laryngology. 2022;279: 3477–3484. doi: 10.1007/s00405-021-07148-y 34716806 PMC8556789

[14] Hummel T, Rosenheim K, Konnerth C-G, Kobal G. Screening of Olfactory Function with a Four-Minute Odor Identification Test: Reliability, Normative Data, and Investigations in Patients with Olfactory Loss. Annals of Otology, Rhinology & Laryngology. 2001;110: 976–981. doi: 10.1177/000348940111001015 11642433

[15] Jackman AH, Doty RL. Utility of a Three-Item Smell Identification Test in Detecting Olfactory Dysfunction. Laryngoscope. 2005;115: 2209–2212. doi: 10.1097/01.mlg.0000183194.17484.bb 16369168

[16] Doty RL, Marcus A, William Lee W. Development of the 12-Item Cross-Cultural Smell Identification Test(CC-SIT). Laryngoscope. 1996;106: 353–356. doi: 10.1097/00005537-199603000-00021 8614203

[17] Trecca EMC, Cassano M, Longo F, Petrone P, Miani C, Hummel T, et al. Results from psychophysical tests of smell and taste during the course of SARS-CoV-2 infection: a review. Acta Otorhinolaryngologica Italica. 2022;42: S20–S35. doi: 10.14639/0392-100X-suppl.1-42-2022-03 35763272 PMC9137382

[18] Le Bon S-D, Pisarski N, Verbeke J, Prunier L, Cavelier G, Thill M-P, et al. Psychophysical evaluation of chemosensory functions 5 weeks after olfactory loss due to COVID-19: a prospective cohort study on 72 patients. European Archives of Oto-Rhino-Laryngology. 2021;278: 101–108. doi: 10.1007/s00405-020-06267-2 32754871 PMC7402072

[19] Schepens EJA, Boek WM, Boesveldt S, Stegeman I, Stokroos RJ, Kamalski DMA. COCOS trial: *CO* rticosteroids for *CO* VID-19-induced loss of *S* mell–protocol for a single-centred, double-blind, randomised, placebo-controlled trial. BMJ Open. 2022;12: e060416. doi: 10.1136/bmjopen-2021-060416 35948382 PMC9378948

[20] Schepens EJA, Blijleven EE, Boek WM, Boesveldt S, Stokroos RJ, Stegeman I, et al. Prednisolone does not improve olfactory function after COVID-19: a randomized, double-blind, placebo-controlled trial. BMC Med. 2022;20: 445. doi: 10.1186/s12916-022-02625-5 36384737 PMC9667850

[21] Schepens EJA, Boek WM, Boesveldt S, Stegeman I, Stokroos RJ, Kamalski DMA. COCOS trial: *CO* rticosteroids for *CO* VID-19-induced loss of *S* mell–protocol for a single-centred, double-blind, randomised, placebo-controlled trial. BMJ Open. 2022;12: e060416. doi: 10.1136/bmjopen-2021-060416 35948382 PMC9378948

[22] Steven mcGee. Evidence-Based Physical Diagnosis. Saunders Elsevier, 4th Edition. 2018.

[23] Boscolo-Rizzo P, Polesel J, Spinato G, Menegaldo A, Fabbris C, Calvanese L, et al. Predominance of an altered sense of smell or taste among long-lasting symptoms in patients with mildly symptomatic COVID-19. Rhinology journal. 2020;58: 524–525. doi: 10.4193/Rhin20.263 32683438

[24] Boscolo-Rizzo P, Hummel T, Hopkins C, Dibattista M, Menini A, Spinato G, et al. High prevalence of long-term olfactory, gustatory, and chemesthesis dysfunction in post-COVID-19 patients: a matched case-control study with one-year follow-up using a comprehensive psychophysical evaluation. Rhinology journal. 2021;0: 0–0. doi: 10.4193/Rhin21.249 34553706

[25] Oleszkiewicz A, Schriever VA, Croy I, Hähner A, Hummel T. Updated Sniffin’ Sticks normative data based on an extended sample of 9139 subjects. European Archives of Oto-Rhino-Laryngology. 2019;276: 719–728. doi: 10.1007/s00405-018-5248-1 30554358 PMC6411676

[26] Sorokowska A, Albrecht E, Haehner A, Hummel T. Extended version of the “Sniffin’ Sticks” identification test: Test–retest reliability and validity. J Neurosci Methods. 2015;243: 111–114. doi: 10.1016/j.jneumeth.2015.01.034 25677404

[27] Gudziol V, Lötsch J, Hähner A, Zahnert T, Hummel T. Clinical significance of results from olfactory testing. Laryngoscope. 2006;116. doi: 10.1097/01.mlg.0000234915.51189.cb 17003712

[28] Bossuyt PM, Reitsma JB, Bruns DE, Gatsonis CA, Glasziou PP, Irwig L, et al. STARD 2015: an updated list of essential items for reporting diagnostic accuracy studies. BMJ. 2015; h5527. doi: 10.1136/bmj.h5527 26511519 PMC4623764

[29] Hummel T, Pfetzing U, Lötsch J. A short olfactory test based on the identification of three odors. J Neurol. 2010;257: 1316–1321. doi: 10.1007/s00415-010-5516-5 20232208

[30] Oleszkiewicz A, Schriever VA, Croy I, Hähner A, Hummel T. Updated Sniffin’ Sticks normative data based on an extended sample of 9139 subjects. European Archives of Oto-Rhino-Laryngology. 2019;276: 719–728. doi: 10.1007/s00405-018-5248-1 30554358 PMC6411676

[31] Hummel T, Whitcroft KL, Andrews P, Altundag A, Cinghi C, Costanzo RM, et al. Position paper on olfactory dysfunction. Rhinology journal. 2017;54: 1–30. doi: 10.4193/Rhino16.248 29528615

